# A prospective double-blinded randomised control trial comparing robotic arm-assisted functionally aligned total knee arthroplasty versus robotic arm-assisted mechanically aligned total knee arthroplasty

**DOI:** 10.1186/s13063-020-4123-8

**Published:** 2020-02-18

**Authors:** Babar Kayani, Sujith Konan, Jenni Tahmassebi, Sam Oussedik, Peter D. Moriarty, Fares S. Haddad

**Affiliations:** Department of Trauma and Orthopaedic Surgery, University College Hospital, 235 Euston Road, Fitzrovia, London, NW1 2BU UK

## Abstract

**Background:**

Total knee arthroplasty (TKA) with mechanical alignment (MA) aims to achieve neutral limb alignment in all patients, whereas TKA with functional alignment (FA) aims to restore native, patient-specific anatomy and knee kinematics by manipulating bone resections and fine-tuning implant positioning. The objective of this study is to determine the optimal alignment technique in TKA by comparing patient satisfaction, functional outcomes, implant survivorship, complications, and cost-effectiveness in MA TKA versus FA TKA. Robotic technology will be used to execute the planned implant positioning and limb alignment with high-levels of accuracy in all study patients.

**Methods and analysis:**

This prospective double-blinded randomised control trial will include 100 patients with symptomatic knee osteoarthritis undergoing primary robotic arm-assisted TKA. Following informed consent, patients will be randomised to MA TKA (the control group) or FA TKA (the investigation group) at a ratio of 1:1 using an online random number generator. Blinded observers will review patients at regular intervals for 2 years after surgery to record predefined study outcomes relating to postoperative rehabilitation, clinical progress, functional outcomes, accuracy of implant positioning and limb alignment, gait, implant stability, cost-effectiveness, and complications. A superiority study design will be used to evaluate whether FA TKA provides superior outcomes compared to MA TKA. Primary and secondary objectives will be used to quantify and draw inferences on differences in the efficacy of treatment between the two groups. Intention-to-treat and per-protocol population analysis will be undertaken. The following statistical methods will be employed to analyse the data: descriptive statistics, independent *t* test, paired *t* test, analysis of variance, Fisher exact test, chi-square test, and graphical displays. Ethical approval was obtained from the London-Surrey Research Ethics Committee, UK. The study is sponsored by University College London, UK.

**Discussion:**

This is the first study to describe the use of robotic technology to achieve FA TKA, and the only existing clinical trial comparing robotic MA TKA versus robotic FA TKA. The findings of this study will enable an improved understanding of the optimal alignment technique in TKA for achieving high-levels of patient satisfaction, improving functional outcomes, increasing implant survivorship, improving cost-effectiveness, and reducing complications.

**Registration:**

Clinical Trials.gov, NCT04092153. Registered on 17 September 2019.

## Background

Total knee arthroplasty (TKA) is an established and highly effective treatment for patients with symptomatic end-stage knee osteoarthritis. The procedure is performed in over 90,000 patients per year in the UK [1]. Middle- to long-term follow-up studies have shown good clinical outcomes following TKA [20, 22, 39], and the 10-year revision rate for cemented, unconstrained, fixed bearing TKA is 3% [1]. Despite these results, there is a higher incidence of patient dissatisfaction compared to total hip arthroplasty, with up to 20% of patients reporting dissatisfaction in an otherwise uncomplicated TKA [5, 9, 10, 35]. The exact aetiology of this is not clear but recent studies have shown one possible reason to be suboptimal limb alignment, which may adversely affect postoperative knee biomechanics and kinematic function [4, 7, 9–11, 35, 44]. Conceptually, an improved understanding and execution of the optimal alignment in TKA may help to increase patient satisfaction, improve functional outcomes and reduce long-term complications.

Total knee arthroplasty with mechanical alignment (MA) aims to achieve neutral alignment of the limb. This is achieved by placing implants perpendicular to the mechanical axis of the femur and tibia, and externally rotating the femoral component to obtain a rectangular, balanced flexion-extension gap, which also aids patella tracking [11]. Measured bone resections or gap balancing techniques with controlled periarticular soft tissue releases help to achieve balanced flexion-extension gaps and restore equal mediolateral soft tissue tension. The principle of neutral mechanical alignment is to distribute load evenly across the implants, which provides a mechanical advantage in flexion and limits asymmetrical bearing surface wear [44]. However, recent studies have shown that there are large variations in native knee anatomy with only 5–5.5% of the general population having natural neutral mechanical alignment [4, 6]. Therefore, in the large majority of patients undergoing MA TKA, the knee is forced into an unnatural position with resultant changes in knee biomechanics that alter the native femoral flexion axis, ligament tension, quadriceps function, patella tracking and overall knee kinematics [4, 6, 17, 18].

Total knee arthroplasty with functional alignment (FA) aims to restore joint line height, preserve native obliquity, and achieve balanced flexion-extension gaps with equal mediolateral soft tissue tension by manipulating bone resections and fine-tuning implant positioning. Conceptually, FA TKA reduces the need for intraoperative periarticular soft tissue releases while restoring the patient’s native pre-arthritic knee kinematics. This technique is a modification of TKA with kinematic alignment, in which bone resections and implant positioning are undertaken to restore the patient’s natural distal and femoral joint lines, tibial joint line and limb alignment. Patient-specific implants, computer navigation and three-dimensional printed cutting blocks have been used to help achieve kinematic alignment in TKA. Studies have demonstrated that TKA with kinematic alignment reproduces more natural knee kinematics including medial pivot movement and femoral rollback compared to MA TKA [14, 34, 15, 26, 29]. Preserving patient-specific alignment and knee kinematics in TKA with kinematic alignment may also decrease the risk of common peroneal nerve palsy, which is associated with forcing the limb into neutral alignment with extensive bone resections and periarticular releases in MA TKA [23, 25]. Early clinical and functional outcome studies have reported promising outcomes in TKA with kinematic alignment [14, 27, 28], but results of longer-term studies have yet to be published.

There is no uniform consensus on the optimal alignment technique for TKA [8, 12, 16, 19, 30, 33, 36–38, 41]. Some studies have shown improved clinical outcomes with TKA with kinematic alignment compared to MA TKA at short-term follow-up, while other systematic reviews and meta-analyses have shown no difference in outcomes between the two alignment techniques [14, 27, 28, 34, 44]. The main limitations of these existing studies are that different implant designs were used within each treatment group, manually positioned cutting blocks with poor reproducibility were used to achieve the planned limb alignment, intraoperative limb alignment was not assessed, and limited data on functional outcomes or implant survivorship were reported. It is possible to improve on these existing studies by assessing a more comprehensive range of validated clinical and functional outcome measures, blinding both patients and observers recording outcomes, and using radiosteriometric analysis (RSA) to assess implant micromotion for long-term implant survivorship [32, 42, 43]. Importantly, FA TKA offers an avenue for achieving patient-specific kinematics with balanced flexion-extension gaps and equal mediolateral soft tissue tension by manipulating bone resections and fine-tuning implant positioning, while limiting the need for periarticular soft tissue releases. Robotic technology also offers an avenue for executing the planned MA TKA or FA TKA with greater accuracy and reduced outliers. The findings of this study will enable an improved understanding of the optimal alignment technique in TKA for achieving high levels of patient satisfaction, improving functional outcomes, increasing implant survivorship, improving cost-effectiveness and reducing complications.

## Methods/design

### Objectives

The primary objective of this study is to compare the total Western Ontario and McMaster Universities Arthritis Index (WOMAC) score in MA TKA versus FA TKA at 2 years after surgery. As FA TKA enables improved restoration of native, patient-specific knee kinematics [15, 26, 29], the study hypothesis is that total WOMAC scores will be superior in patients undergoing FA TKA compared to MA TKA at 2 years follow-up.

The secondary objectives are to compare the following outcomes between the two treatment groups:

1. Accuracy of implant positioning and limb alignment
2. Surgical efficiency
3. Postoperative functional rehabilitation
4. Functional outcomes
5. Quality of life
6. Implant migration
7. Gait
8. Resource use and cost-effectiveness
9. Complications

### Trial design

**Fig. 1:**
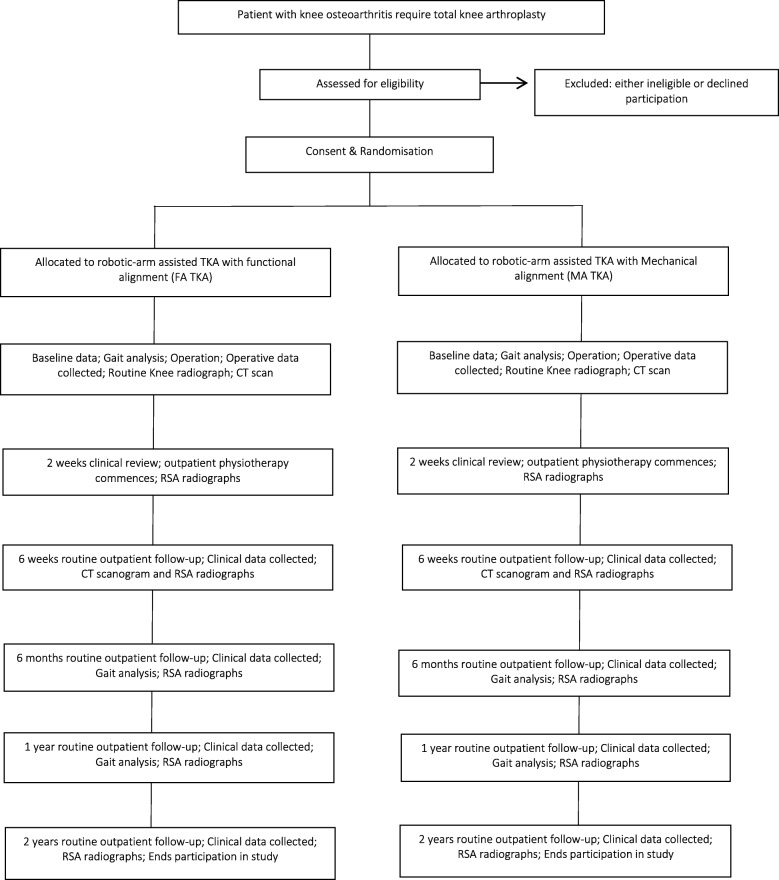
Patient enrolment flow chart. CT computerised tomography, RSA radiosteriometric analysis, TKA total knee arthroplasty

**Fig. 2:**
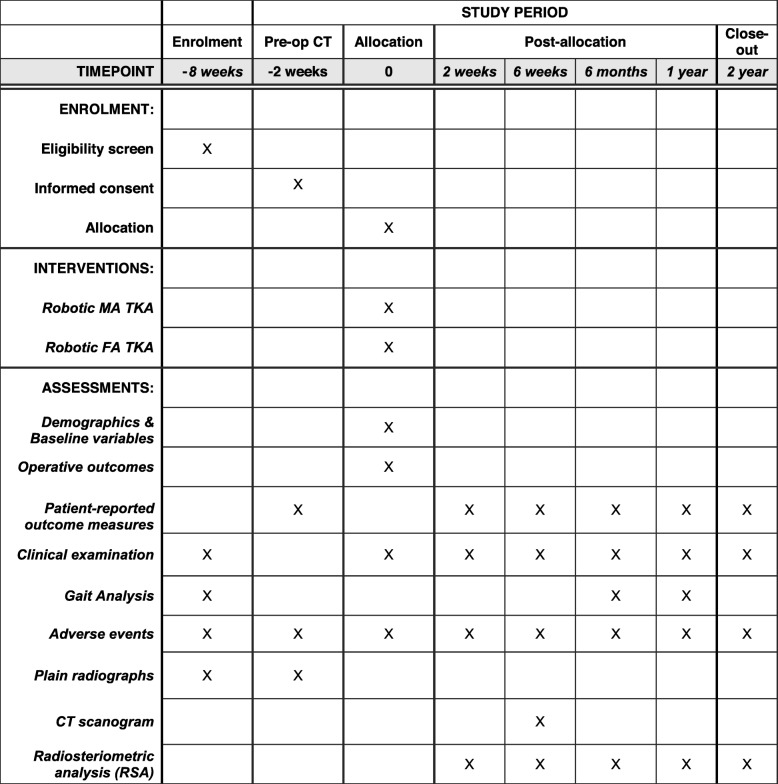
Schedule of enrolment, interventions, and assessments for all study patients*.* Legend: *CT* computerised tomography, *FA TKA* total knee arthroplasty with functional alignment, *MA TKA* total knee arthroplasty with mechanical alignment

This study is a prospective, single-centre, double-blinded, randomised control trial. The study will be undertaken in the Department of Trauma and Orthopaedics, University College Hospital, London, UK. The study will include 100 patients randomly allocated to either MA TKA (the control group) or FA TKA (the investigation group). All patients will undergo robotic arm-assisted TKA to improve the accuracy of achieving the planned implant positioning and limb alignment. The study commenced patient recruitment in December 2018 and is expected to complete patient recruitment in December 2020. All patients will be followed up for 2 years after surgery and therefore the anticipated completion date for the study is December 2022. The study is sponsored by University College London, UK. The patient enrolment flowchart is presented in Fig. 1. The schedule of enrolment, interventions, and assessments for all study patients is shown in Fig. 2. This study followed the Standard Protocol Items: Recommendations for Interventional Trials (SPIRIT) (Additional file 1).

### Eligibility criteria

The inclusion criteria for this study are as follows: 1) the participant has symptomatic knee osteoarthritis requiring primary TKA; 2) the participant is fit for surgical intervention following a review by the surgeon and anaesthetist; 3) the participant is aged between 18 and 80 years at the time of surgery; 4) the participant is able to give informed consent and agrees to comply with the postoperative review programme; and 5) the participant has sufficient mobility to attend follow-up clinics. The exclusion criteria for this study are as follows: 1) the participant is undergoing revision surgery or second-stage TKA; 2) the participant is not suitable for study implants (e.g. requires a constrained prosthesis); 3) the participant is immobile or has another neurological condition affecting musculoskeletal function; 4) the participant is already enrolled on another concurrent clinical trial; 5) the participant is unable or unwilling to sign the informed consent form specific to this study; and 6) the participant is unable to attend the study follow-up programme.

### Recruitment

Participants will be recruited from the orthopaedic outpatient clinic at University College Hospital, London, UK. All patients will be screened by the clinical team (orthopaedic consultant surgeon, clinical research fellow, and orthopaedic registrar) for study participation based on the predefined inclusion and exclusion criteria listed above. Patients that fulfil the eligibility criteria and express an interest in participating in the study will be provided with an Ethics Committee-approved patient information sheet. This provides details about the study, treatment, follow-up and contact details for further information. All members of the clinical team are familiar with the study and will address any preliminary questions about the study. Details of those patients expressing an interest to participate in the study will be recorded in the patient contact form and forwarded to the research physiotherapist. The research physiotherapist will telephone the patient 4 weeks after this consultation to discuss any further questions and confirm if the patient would like to participate in the study.

### Consent

Informed consent will be obtained by the chief investigator or principal investigator when the patient attends for the preoperative planning computerised tomography (CT) scan. This is 6 weeks after the outpatient consultation for agreement to TKA and 2 weeks before surgery. It is important to the data collection scheme that patients are able to follow commands and read and interpret questions via questionnaires. For those who cannot hear, read or understand English, an interpreter will be provided. The operating surgeon will use the preoperative CT scan to create a patient-specific computer-aided design model and create a surgical plan for executing both MA TKA and FA TKA in all study patients.

### Allocation

After informed consent has been obtained, the research physiotherapist will randomise the patient into one of the two groups using an online random number generator (www.random.org). A number from 1 to 100 will be randomly generated and will allocate a patient to one of the two arms of the study: 1–50 inclusive for the control group, 51–100 inclusive for the investigation group. The research physiotherapist will perform the randomisation procedure and store the designated treatment group for each patient on a password-encrypted file on the hospital computer. The operating surgeon will have this information communicated to him on the morning of surgery.

### Surgical intervention

In patients undergoing MA TKA, femoral and tibial bone implant positioning will be used to achieve neutral limb alignment. In the coronal plane, femoral implant positioning will be set at 5–7 valgus in relation to the anatomical axis of the femur. In the sagittal plane, femoral component positioning will be set at 0–5° of flexion to optimise implant positioning while preventing notching. In the axial plane, the femoral component will be aligned to the surgical transepicondylar axis, which is approximately 3° externally rotated to the posterior condylar axis [2, 3]. The size of the femoral implant will be selected using posterior referencing with the largest size that does not overhang the femur, notch the anterior femur, or overhang the mediolateral bone edges, and avoids overstuffing the patellofemoral joint. The femoral implant will be positioned at the centre of the mediolateral cortical bone edges. In the coronal plane, tibial implant position will be aligned to the tibial mechanical axis. In the sagittal plane, tibial implant position will be set to 0–3° of posterior tibial slope. In the axial plane, tibial implant will be positioned at 0–5° of external rotation to Akagi’s line [2, 3, 45]. Tibial implant size will be selected with the largest size that does not overhang the anteroposterior or mediolateral bone coverage. The implant will be positioned in the centre between the anteroposterior and mediolateral cortical bone edges.

In patients undergoing FA TKA, implants will be positioned to optimise soft tissue tension through achieving balanced flexion-extension gaps and equal mediolateral soft tissue tension by altering bone resections and implant positions rather than through soft tissue releases. This will be achieved when possible within strict alignment limits, and where not achievable because of the magnitude of a fixed deformity by balancing after bone cuts with limited soft tissue releases. The preoperative surgical plan will be used to fix a specific point on the tibia and the gaps balanced to restore the obliquity of the native joint line. In the coronal plane, femoral implant positioning will be modified from a starting point of 0° to the mechanical axis to balance the extension gap. In the sagittal plane, femoral component positioning will be set to optimise component sizing while avoiding notching by flexing up to 5°. In the axial plane, the femoral component will be aligned to the surgical transepicondylar axis and modified by up to 3° to balance the flexion gap. The size of the femoral implant will be selected using posterior referencing with the smallest size that does not overhang the femur, notch the anterior femur, or overhang mediolateral bone edges, and avoids overstuffing the patellofemoral joint. The femoral implant will be positioned at the centre of the mediolateral cortical bone edges, favouring a lateral position if necessary. In the coronal plane, tibial implant position will be aligned to the tibial mechanical axis and then modified to balance flexion and extension gaps by up to 3° of varus. Valgus tibial position will be avoided. In the sagittal plane, tibial implant position will be set to match the patient’s native posterior tibial slope, modified to balance the flexion gap if necessary. In the axial plane, the tibial implant will be positioned using Akagi’s line [2, 3, 45]. Tibial implant size will be selected with the largest size that does not overhang the anteroposterior and mediolateral bone coverage while achieving the correct rotation. The implant will be positioned in the centre between the anteroposterior and mediolateral cortical bone edges.

All operative procedures will be undertaken using the Mako robotic arm interactive orthopaedic system (Stryker Limited, Kalamazoo, MI, USA) under the direct supervision of one arthroplasty surgeon (FSH). The cemented Stryker Triathlon (Stryker Navigation, Kalamazoo, MI, USA) cruciate-retaining knee system with asymmetrical patellar resurfacing will be used in both groups. All bone resections, implant positioning and limb alignment will be within regulatory approval for the Triathlon cruciate-retaining knee system. Overall limb alignment, defined as the sum of the femoral and tibial coronal rotations, will range from 3° of varus to 3° of valgus.

### Outcomes

All study patients will undergo review by two blinded observers (one orthopaedic registrar and one clinical research fellow) at 2 weeks, 6 weeks, 6 months, 1 year and 2 years following surgery. During these follow-up times, predefined clinical, functional and radiological outcomes will be recorded by these observers using case report forms. The following outcomes will be recorded in all study patients:

1. Accuracy of implant positioning and limb alignment as assessed using CT scans of the knee joint performed postoperatively at 6 weeks.
2. Operating time (minutes)
3. Time to hospital discharge (hours)
4. Analgesia requirements during inpatient admission and postoperatively at 6 weeks, 6 months, 1 year and 2 years
5. Patient-reported outcome measures including Forgotten Joint Score (FJS), Oxford Knee Score (OKS), short-form health survey of 12 items (SF-12), Knee Injury and Osteoarthritis Outcome Score (KOOS), WOMAC, University of California at Los Angeles score and University College Hospital functional knee score preoperatively and postoperatively at 6 weeks, 6 months, 1 year and 2 years
6. Health-related quality of life as measured using the European Quality of Life questionnaire with five dimensions for adults (EQ-5D) preoperatively and postoperatively at 6 weeks, 6 months, 1 year and 2 years
7. Mobilisation distance (metres) and use of mobility aids during inpatient admission and postoperatively at 6 weeks, 6 months, 1 year and 2 years
8. Range of movement (degrees) in knee joint during inpatient admission and postoperatively at 6 weeks, 6 months, 1 year and 2 years
9. Femoral and tibial implant early migration as assessed using RSA performed postoperatively at 2 weeks, 6 weeks, 6 months, 1 year, and 2 years
10. Gait analysis performed postoperatively at 6 months and 1 year using an instrumented treadmill with force plates
11. Resource use and cost-effectiveness, including comparisons between the two treatment groups relating to operating time, theatre efficiency, equipment and sterilisation costs, analgesia requirements, inpatient rehabilitation, time to discharge, outpatient follow-up, additional imaging costs and need for further surgery.
12. Complications

The FJS, University of California at Los Angeles knee score, WOMAC, OKS, KOOS, SF-12 and EQ-5D are validated tools for the clinical assessment of patients after knee arthroplasty [21, 24, 31]. In addition, the blinded observer will record the University College Hospital functional knee score to assess overall pain, function and mobility. All study patients will undergo gait analysis using an instrumented treadmill with force plates (Kistler Gaitway, Kistler Instrument Corporation, Amherst, NY, USA) on a level platform. Gait analysis will be performed at the patient’s self-selected comfortable speed and maximum speed without running. Vertical ground reaction forces and spatiotemporal data will be obtained from force plates built into the treadmill. RSA radiographs will be performed at regular postoperative follow-up intervals to quantify motion between the implant and host bone, which is highly predictive of long-term implant survival [32, 40].

### Blinding

All patients and clinical staff recording postoperative study outcomes will remain blinded to the treatment group. Study patients will be identifiable with a unique study number. Only the research physiotherapist will have the key to identify individual patients and their respective treatment arm. Any documents related to the study will be archived directly at the study site by the research physiotherapist within a secure filing cabinet in a locked research office. This office has swipe card access with onsite security and 24-h closed-circuit television surveillance. Patient data will be logged electronically using each patient’s unique identification number with computer software on an encrypted, password-protected research computer.

### Sample size

Using data from a previous study recording functional outcomes, the mean WOMAC score at 2 years using MA TKA was 26 (standard deviation 22.6) and using TKA with kinematic alignment was 15 (standard deviation 20.3) [14]. Using a two-tailed, two-sample *t* test with an effect size of 0.35, power of 90% with significance level of 5%, and accounting for an expected drop-out rate of 10% during the 2-year follow-up period, the study requires 100 patients to detect a minimal clinically important difference of 11 points in the total WOMAC score between the two treatment groups [13].

### Statistical analysis

The analysis of the per-protocol population will be considered the primary analysis. The differences between the MA TKA and FA TKA groups will be analysed by calculating the difference from baseline per patient, and a two-sided confidence interval for the difference between the changes from baseline values will be calculated. This confidence interval will cover the true difference in the percentage change from baseline with a probability of 95%. The following statistical methods will be employed to analyse the data: descriptive statistics, independent *t* test, paired *t* test, analysis of variance, Fisher exact test, chi-square test and graphical displays. Assumptions of normality will be tested with the D’Agostino test. Assumptions of homogeneity of variance will be tested with Levene’s test. If the distributional assumptions are (severely) violated, non-parametric techniques such as the Mann–Whitney test will be employed. In the event that FA TKA is converted to MA TKA intraoperatively, analysis will be performed using the intention-to-treat population and the treatment actually received by the patients. Intraoperative conversion from FA TKA to MA TKA will be documented and presented and published as part of the study. Statistical significance is set at a *P* value <0.05 for all analyses and all statistical analyses will be performed using SPSS software version 25 (SPSS Inc., Chicago, IL, USA). The Bonferroni correction will be used to adjust *P* values to reduce the risk of type I error with performing multiple statistical comparisons.

### Adverse events

Adverse events are defined as any untoward medical occurrence in a patient or study participant that does not necessarily have a causal relationship with the procedure involved. A serious adverse event (SAE) is an adverse event that results in hospitalisation or prolongation of existing hospitalisation, persistent or significant disability or incapacity, life-threatening clinical sequelae, or death. All SAEs during the protocol treatment will be reported directly to the sponsor using the SAE web form. The chief investigator will also assess the SAE for severity, causality, seriousness and expectedness using pre-existing criteria provided by the sponsor and will inform the Data Safety Monitoring Board (DSMB) within 3 days of the initial observation of the event. The protocol treatment period is defined as the period from the day that the first study patient is recruited into the trial to the day that the final study patient has completed 2 years follow-up. The chief investigator will also inform the London-Surrey Research Ethics Committee and local Health Research Authority within 3 days of the SAE taking place. Safety aspects of the study are closely monitored by the sponsor and DSMB using unblinded data for its judgment. In cases where the SAE arises due to a problem with the robotic device, Stryker Limited will also be notified within 2 days of the event taking place. The chief investigator will record the following: onset date, complete description of the event, severity, duration, action taken and outcome for each SAE. The chief investigator will also provide regular updates of all SAEs to the London-Surrey Research Ethics Committee, local Health Research Authority, DSMB, and sponsor.

### Data management

On-site monitoring visits shall occur throughout the course of the clinical study by the chief investigator. The chief investigator shall permit and assist the sponsor (should they chose to monitor the study) to carry out verification of all study forms against data in the source documents, which shall occur as per the departmental policy for undertaking such activities. University College Hospital recognises that there is an obligation to archive study-related documents at the end of the study. The study master file will be archived at University College London in accordance with the University College Hospital Standard Operating Procedure for Archiving of Investigator Site File and Pharmacy Site File. It will be archived for a minimum of 5 years from the study end, and for no longer than 30 years from the study end.

### End-of-protocol treatment

Reasons for going off study protocol include:

1. Completion of last follow-up visit 2 years after surgery
2. Patient non-compliance or withdrawal (the reason for discontinuation will be recorded in the case report form)
3. Intercurrent death

All patients included in this study are free to withdraw from the study at any time without compromise to their future treatment. On withdrawal, patients will revert to the standard follow-up regimen for routine (non-study) TKA at the study site. The end-of-study form will be completed and the reason for withdrawal documented. This form will also be completed if the patient is lost to follow-up or dies during the course of the study. Data to the point of discontinuation will be used for analysis.

### Monitoring

The chief investigator will monitor the progress of the clinical study in the form of monthly research meetings for those involved in the trial. The chief investigator will be responsible for the day-to-day monitoring and management of the study. The University College Hospital/University College London/Joint Research Office, on behalf of University College London as Sponsor, will monitor and conduct random audits on a selection of studies in its clinical research portfolio. Monitoring and auditing will be conducted in accordance with the Department of Health Research Governance Framework for Health and Social Care (April 2005), and in accordance with the sponsor’s monitoring and audit policies and procedures. As per the protocol, the principal investigator will email the sponsor twice yearly with the following information: delegation log, adverse event log, deviation log, and any annual progress reports sent to the Ethics Committee.

### Peer review

The study protocol was reviewed by two external reviewers. The suggestions and recommendations for improvement to the study design were implemented. The reviewers and sponsor reviewed the revised protocol documents and confirmed that all queries and suggestions had been fully addressed.

## Discussion

The concept of MA TKA is to distribute load evenly across the components to optimise implant survivorship and balance forces through the periarticular soft tissue envelope for proper functioning of the knee joint. However, in the majority of patients this forces the knee into an unnatural position with altered knee kinematics through the arc of flexion [4, 6, 17, 18]. FA TKA aims to restore joint line height, preserve native obliquity, and achieve balanced flexion-extension gaps with equal mediolateral soft tissue tension by manipulating bone resections and fine-tuning implant positioning, which reduces the need for soft tissue releases. To our knowledge, this prospective randomised control trial is the first study to compare MA TKA with FA TKA. Robotic technology will be used in both treatment groups, which will enable accurate execution of the preoperative surgical plan and help preserve the double-blinded nature of this study. Furthermore, RSA will be used to compare micromotion and implant survivorship between the two treatment groups. The findings of this study will enable an improved understanding of the optimal alignment technique in TKA for achieving high-levels of patient satisfaction, improving functional outcomes, increasing implant survivorship, improving cost-effectiveness and reducing complications.

## Trial status

This is protocol version 3.0, 1 June 2018. Patient recruitment started on 28 December 2018. The estimated date for completion of recruitment is 28 December 2020. The estimated date for completion of the final follow-up is 28 December 2012.

## Supplementary information

**Additional file 1.** Standard Protocol Items: Recommendations for Interventional Trials (SPIRIT) 2013 checklist—recommended items to address in a clinical trial protocol and related documents.

## Data Availability

The datasets used and/or analysed during the current study are available from the corresponding author on reasonable request.

## Abbreviations

DSMB: Data Safety Monitoring Board
EQ-5D: European Quality of Life questionnaire with five dimensions for adults
FA: Functional alignment
FJS: Forgotten Joint Score
KOOS: Knee Injury and Osteoarthritis Outcome Score
MA: Mechanical alignment
OKS: Oxford Knee Score
RSA: Radiosteriometric analysis
SAE: Serious adverse event
SF-12: Short-form health survey of 12 items
TKA: Total knee arthroplasty
WOMAC: Western Ontario and McMaster Universities Arthritis Index
