# Correction: A prospective double-blinded randomised control trial comparing robotic arm-assisted functionally aligned total knee arthroplasty versus robotic arm-assisted mechanically aligned total knee arthroplasty

**DOI:** 10.1186/s13063-026-09948-1

**Published:** 2026-08-13

**Authors:** Babar Kayani, Sujith Konan, Jenni Tahmassebi, Sam Oussedik, Peter D. Moriarty, Fares S. Haddad

**Affiliations:** Department of Trauma and Orthopaedic Surgery, University College Hospital, 235 Euston Road, Fitzrovia, London, NW1 2BU UK

**Correction: Trials 21, 194 (2020)**

**https://doi.org/10.1186/s13063-020-4123-8**

Following the publication of the original article [1], we were notified that during the conduct of the trial a discrepancy was identified between the stated sample size and the result of the calculation under the parameters cited, and the sample size was recalculated at that time.

Yeo et al. [2] reported outcomes from a prospective randomised study of robotic-assisted TKA comparing mechanical and kinematic alignment at a mean follow-up of 8 years. Mean total WOMAC score was 20.4 ± 1.8 in the mechanical alignment group and 19.3 ± 1.9 in the kinematic alignment group, giving a pooled standard deviation of 1.85. Using a two-tailed, two-sample t-test with 80% power and significance level of 5%, 45 patients per group are required to detect a between-group difference of 1.1 points. Accounting for 10% attrition over the two-year follow-up, 100 patients were recruited.

The clinical meaningfulness of this sample size is independently supported by anchor-based psychometric data reported by Clement et al. [3, 4] in 2,589 patients undergoing primary TKA, which established a minimum clinically important difference (MCID) of 10 points, a minimum important change (MIC) of 16.5 points, and a minimum detectable change at 95% confidence of 11.6 points for the total WOMAC score, with a reliability coefficient of 0.921 and standard error of measurement of 4.19 points. Applying each of these thresholds as the target detectable difference with 80% power and two-sided alpha of 0.05, the required sample size ranges from 13 to 35 patients per group. The enrolled sample of 100 patients exceeds the requirement under every clinically established threshold.

The authors apologise for the error in the original publication.

The original article has been corrected.

## References

[1] Kayani B, Konan S, Tahmassebi J, et al. A prospective double-blinded randomised control trial comparing robotic arm-assisted functionally aligned total knee arthroplasty versus robotic arm-assisted mechanically aligned total knee arthroplasty. Trials. 2020;21:194. 10.1186/s13063-020-4123-8. 32070406 10.1186/s13063-020-4123-8 PMC7027302

[2] Yeo JH, Seon JK, Lee DH, Song EK. No difference in outcomes and gait analysis between mechanical and kinematic knee alignment methods using robotic total knee arthroplasty. Knee Surg Sports Traumatol Arthrosc. 2019;27:1142–7. 10.1007/S00167-018-5133-X/TABLES/5. Cited 2026 Jun 27. Springer Verlag. 30220048 10.1007/s00167-018-5133-x

[3] Clement ND, Bardgett M, Weir D, Holland J, Gerrand C, et al. What is the minimum clinically important difference for the WOMAC index after TKA? Clin Orthop Relat Res. 2018;476:2005–14. 10.1097/CORR.0000000000000444. 30179956 10.1097/CORR.0000000000000444 PMC6259858

[4] Clement ND, Bardgett M, Weir D, Holland J, Gerrand C, Deehan DJ. Erratum to: What is the minimum clinically important difference for the WOMAC index after TKA? Clin Orthop Relat Res. 2020;478:922. 10.1097/CORR.0000000000001156. [cited 2026 Jun 27]. 10.1097/CORR.0000000000001156 PMC7282586 32187100

